# 2063. Elucidating the Gram-Positive Selective Spectrum Activity of Ibezapolstat; Secondary Analysis from the phase 2a trial

**DOI:** 10.1093/ofid/ofad500.133

**Published:** 2023-11-27

**Authors:** Chris Lancaster, Jacob McPherson, M Jahangir Alam, Khurshida Begum, Md Ekramul Karim, Chenlin Hu, Eugenie Basseres, Kevin W Garey

**Affiliations:** University of Houston, Houston, Texas; University of Houston College of Pharmacy, Houston, Texas; Department of Pharmacy Practice and Translational Research, University of Houston College of Pharmacy, Houston, Texas, USA, Houston, Texas; Department of Pharmacy Practice and Translational Research, University of Houston College of Pharmacy, Houston, Texas, USA, Houston, Texas; Department of Pharmacy Practice and Translational Research, University of Houston College of Pharmacy, Houston, Texas, USA, Houston, Texas; University of Houston, Houston, Texas; University of Houston College of Pharmacy, Houston, Texas; University of Houston, Houston, Texas

## Abstract

**Background:**

Ibezapolstat (IBZ) is a non-absorbable antimicrobial currently in phase 2 clinical trials for the treatment of *Clostridioides difficile* infection (CDI). *In vitro* and human studies have shown potent activity of IBZ against *C. difficile* but selective activity against other beneficial Gram-positive gut microbiota shown to reduce the risk of recurrent CDI. As the target DNA Pol IIIC enzyme is present in most Gram-positive species, the reasons for this selectivity are unclear. The purpose of this study was to assess the selectivity of IBZ against Gram-positive gut commensal microbiota.

**Methods:**

Using stool samples and microbiome data from the phase 2a CDI study, concentration changes in certain Gram-positive commensals were analyzed by qPCR over time in patients with CDI given IBZ. Gram-positive isolates were cultured from stool, speciated, and MIC determined. AlphaFold2-enabled drug docking was used to assess in silico differences in drug-binding residue sites predictive of IBZ binding across a large range of Gram-positive bacteria isolated from the stool.

**Results:**

From the phase 2a study, relative abundance of Firmicutes and concentrations of certain species (*Clostridium leptum* and *Clostridium coccoides* groups) increased while on IBZ therapy. Thirty-six strains were isolated from Families Clostridiaceae (n=25), Enterococcaceae (n=3), Lachnospiraceae (n=3), and Peptostreptococcaceae (n=5). MIC50/90 values averaged 1.5/3.0 ug/mL (geometric mean) for strains isolated at baseline and early therapy (days 1-3) and 12/126 ug/mL for samples taken later on therapy and three-day follow-up. Two strains isolated at day 30 follow-up were < 2 ug/mL and another was >128 ug/mL. All *C. difficile* strains were IBZ susceptible (MIC<2 ug/mL). Using a protein sequence alignment from more than 500 Firmicute PolC sequences, potential contact points of interest were identified that helped explain the selectivity of Ibezapolstat.

**Conclusion:**

Increased Firmicute abundance and concentration may be explained by selection of non-susceptible Firmicute species during therapy. Distinct IBZ drug-binding residues in the Pol IIIC enzyme may explain this selectivity. Structural biology experiments will confirm these results.

**Disclosures:**

**Eugenie Basseres, PhD**, Accurx: Grant/Research Support **Kevin W. Garey, PharmD, MS**, Acurx: Grant/Research Support|Ferring: Advisor/Consultant|Paratek: Grant/Research Support

